# Travel-associated Antimicrobial Drug–Resistant Nontyphoidal Salmonellae, 2004–2009

**DOI:** 10.3201/eid2004.131063

**Published:** 2014-04

**Authors:** Russell S. Barlow, Emilio E. DeBess, Kevin L. Winthrop, Jodi A. Lapidus, Robert Vega, Paul R. Cieslak

**Affiliations:** Oregon Health Authority, Portland, Oregon USA (R.S. Barlow, E.E. DeBess, P.R. Cieslak);; Oregon Health and Science University, Portland (R.S. Barlow, K.L. Winthrop, J.A. Lapidus);; Oregon State Public Health Lab, Hillsboro, Oregon, USA (R. Vega)

**Keywords:** Salmonella, drug resistance, foodborne diseases, communicable diseases, emerging, bacteria, Asia, travel, nontyphoidal

## Abstract

To evaluate trends in and risk factors for acquisition of antimicrobial-drug resistant nontyphoidal *Salmonella* infections, we searched Oregon surveillance data for 2004–2009 for all culture-confirmed cases of salmonellosis. We defined clinically important resistance (CIR) as decreased susceptibility to ampicillin, ceftriaxone, ciprofloxacin, gentamicin, or trimethoprim/sulfamethoxazole. Of 2,153 cases, 2,127 (99%) nontyphoidal *Salmonella* isolates were obtained from a specific source (e.g., feces, urine, blood, or other normally sterile tissue) and had been tested for drug susceptibility. Among these, 347 (16%) isolates had CIR. The odds of acquiring CIR infection significantly increased each year. Hospitalization was more likely for patients with than without CIR infections. Among patients with isolates that had been tested, we analyzed data from 1,813 (84%) who were interviewed. Travel to eastern or Southeast Asia was associated with increased CIR. Isolates associated with outbreaks were less likely to have CIR. Future surveillance activities should evaluate resistance with respect to international travel.

Each year, nontyphoidal salmonellae (NTS) are responsible for >1 million infections in the United States and an estimated 98 million cases globally (*1*–*4*). Each year in the United States, infections result in an estimated 168,000 physician visits, 19,000 hospitalizations, and 380 deaths at a cost of $US 2.3 billion (*1*–*3*,*5*). Data suggest that 85.6% of NTS infections are foodborne and that the remaining infections occur by the fecal–oral route in human-to-human transmission and zoonotic transmission (*2*). For healthy persons, infections commonly result in self-limiting acute gastroenteritis that resolves without antimicrobial drug therapy. However, antimicrobial drugs can be life saving for immunologically vulnerable populations, such as infants, elderly persons, immunocompromised persons, and persons with invasive infection (*6*–*8*). The drugs most commonly prescribed in developing countries are ampicillin and chloramphenicol; those most commonly prescribed in the United States are trimethoprim/sulfamethoxazole, fluoroquinolones, and cephalosporins (*9*).

In the 1980s, studies demonstrated alarming increases in the prevalence of antimicrobial drug resistance among NTS infections (*10*,*11*). This increase was associated with indiscriminate use of antimicrobial drugs in animal husbandry and in humans (*10*–*12*). A retrospective study conducted during 1996–2001 associated antimicrobial drug resistance with increased disease severity, highlighting the risk to public health (*13*).

During the past decade, few population-level analyses have identified risk factors for acquiring a resistant NTS infection outside of outbreak clusters and retail meat supplies. A recently identified risk factor is international travel (*14*,*15*). Bacteriologic studies from Europe identified differences in resistance among *Salmonella enterica* serotypes Stanley, Concord, and Typhimurium isolated from patients with a history of international travel (*16*–*20*). However, these studies did not estimate the magnitude of or risk for antimicrobial drug–resistant NTS acquisition among international travelers. We hypothesized that international travel is a risk factor for acquisition of a resistant NTS infection. To test our hypothesis, we analyzed surveillance data from Oregon for 2004–2009 and quantified trends in antimicrobial drug resistance, investigated the relationship between resistance and case outcomes, and assessed whether international travel was associated with acquisition of NTS infections with clinically important resistance (CIR).

## Methods

### Reportable Infectious Disease Surveillance in Oregon

The Oregon Health Authority conducts active, laboratory-based surveillance for all cases of NTS infection. Physicians and laboratories are required by law to report laboratory-confirmed and clinically suspected cases of salmonellosis to the patient’s local health department; reports should contain the patient’s date of birth, sex, diagnosis, date of symptom onset, date of specimen collection, and laboratory test results. All *Salmonella* isolates are forwarded to the Oregon State Public Health Laboratory (OSPHL), where they are serotyped. Local health department officials interview patients about hospitalization, clinical outcomes, additional demographic information, and exposure history for the 7 days before illness onset. Risk-factor questions ask about specific travel, human, animal, and high-risk food exposures. International travel was considered a risk factor only if it had taken place in the 30 days before illness onset. Patients with recurrent infection or multiple *Salmonella* isolates (of same serotype within a plausible time frame for the original infection) are interviewed only once, at the time of initial illness onset.

During 2004–2009, the population of Oregon was 3.6–3.8 million persons, which is ≈1.2% of the US population (*21*,*22*). The surveillance system in Oregon is estimated to capture >99% of laboratory-confirmed cases of salmonellosis; however, for every 1 case confirmed, an estimated 25 additional cases are not detected (*2*).

### Antimicrobial Drug Susceptibility Testing

For 2004 and 2005, all confirmed isolates were forwarded to the Oregon State University Veterinary Diagnostic Laboratory for susceptibility testing. From 2006 through 2009, susceptibility testing was performed by OSPHL. All isolates were tested by using broth microdilution to determine MICs for the following 10 antimicrobial agents: ampicillin, ceftriaxone, chloramphenicol, ciprofloxacin, gentamicin, nalidixic acid, nitrofurantoin, sulfamethoxazole, tetracycline, and trimethoprim/sulfamethoxazole. Susceptibilities were determined according to Clinical and Laboratory Standards Institute interpretative criteria (*23*). To ascertain cephalosporin resistance, OSPHL tested isolates for ceftriaxone susceptibility; and the Oregon State University Veterinary Diagnostic Laboratory tested for susceptibility to cefuroxime and cephalothin by using analogous broth microdilution methods. MIC results were dichotomized as susceptible or resistant.

### Analyses

Isolates were included in analyses only if they were cultured from specific specimens, such as feces, urine, or blood or other normally sterile tissues (i.e., cerebrospinal fluid). CIR was defined as resistance to at least 1 of the following: ampicillin, ceftriaxone, ciprofloxacin, gentamicin, or trimethoprim/sulfamethoxazole (*13*). We used the Cochran-Armitage test for trend to analyze NTS case data for 2004–2009 to determine whether the proportion of *Salmonella* isolates with CIR increased significantly. Demographic and exposure risk factors, specifically international travel, were evaluated as risk factors for acquisition of a resistant isolate. The 9 most common *Salmonella* serotypes were fixed (included in all models regardless whether they met the p<0.05 level of significance) in all analyses, and remaining serotypes were grouped as “all other.” Serotype Enterditis is the most frequently isolated serotype in Oregon and was therefore used as the referent for all comparisons.

We sought to evaluate whether resistance was associated with increased disease severity, including hospitalization and invasive infection. Invasive infection was defined as isolation of *Salmonella* from a normally sterile body site or tissue, such as blood (*13*). Multiple logistic regression models were constructed with variables that were significant at the p<0.25 level in unadjusted analyses. *Salmonella* serotype and patient race, age, and year of illness onset were fixed in all models. Other variables were given further consideration according to disease severity or relevance for external validity. Predictor variables significant at p<0.05 were retained in the final model, and adjusted log odds ratios (aORs) were calculated. Model fit was assessed by using the Hosmer–Lemeshow goodness-of-fit test.

All analyses were performed by using SAS version 9.2 (SAS Institute Inc., Cary, NC, USA). Because this study involved more extensive analysis only of data collected routinely as part of public health surveillance, it was not considered human subjects research.

## Results

### Descriptive Epidemiology and Resistance Trends

From 2004 through 2009, a total of 2,255 laboratory-confirmed cases of nontyphoidal salmonellosis were reported in Oregon. In accordance with Oregon law, 2,153 isolates were forwarded to OSPHL, and 2,127 (98.8% of all NTS isolates) were cultured from a specific source and had antimicrobial drug susceptibility testing information (Figure). Of these isolates, 26 (1.2%) were obtained through a nonspecific source, such as lesions or sputum, and were excluded from analysis.

**Figure:**
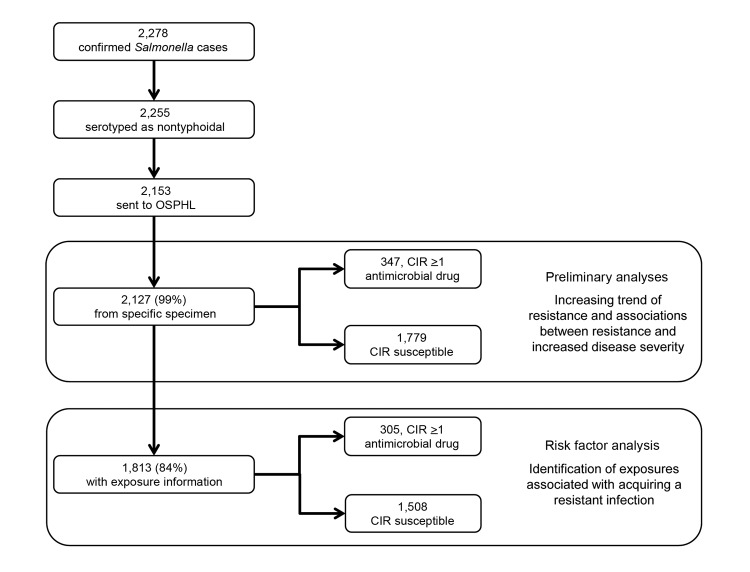
Culture-confirmed salmonellosis cases ascertained by statewide active surveillance and included in analyses, Oregon, USA, 2004–2009. CIR, clinically important resistance; OSPHL, Oregon State Public Health Laboratory.

The most common *Salmonella* serotypes detected were Enteritidis (18.4%), Typhimurium (14.3%), Heidelberg (8.2%), Typhimurium var. Copenhagen (5.1%), and Newport (4.5%). Cases that were part of identified outbreaks represented 24.1% of the cohort; the remaining 75.9% were considered sporadic cases. The median age of patients was 29 years (interquartile range 9–51 years), and 53.1% of patients were female. Information about race was not available for 9%; of patients for whom race was known, 91.8% were white and 9.2% were not white. Similarly, information about ethnicity was not available for 10.5% of patients. Among patients for whom ethnicity was known, 87.4% were not Hispanic and 12.6% were Hispanic. Isolates from 1,213 (57%) patients were susceptible to all antimicrobial drugs screened (pansusceptible), and isolates from 347 (16.3%) had CIR (Table 1). Of the 2,127 patients, 412 (19.4%) were hospitalized and 110 (5.2%) had invasive disease.

**Table 1:** Frequencies of antimicrobial drug resistance among 2,127 *Salmonella* isolates, Oregon, USA, 2004–2009*

Variable	Resistance, no. (%)
Drug	
Ampicillin	285 (13.4)
Ceftriaxone	109 (5.1)
Chloramphenicol	177 (8.3)
Ciprofloxacin	13 (0.6)
Gentamicin	84 (4.0)
Nalidixic acid	135 (6.4)
Nitrofurantoin	283 (13.3)
Sulfamethoxazole	411 (19.3)
Tetracycline	574 (27.0)
Trimethoprim/sulfamethoxazole	60 (2.8)
Resistance profiles	
Pansusceptible	1,213 (57.0)
CIR	347 16.3)

*CIR, clinically important resistance to >1 of the following: ampicillin, ceftriaxone, ciprofloxacin, gentamicin, or trimethoprim/sulfamethoxazole.

The proportion of isolates that were pansusceptible significantly decreased from 69.5% in 2004 to 53.6% in 2009 (p<0.01). CIR did not significantly increase during this study period (p = 0.27). Stratification by serotype revealed that CIR increased among the 3 most common serotypes: Enteritidis (3% to 8%, p = 0.02), Typhimurium (19% to 34%, p = 0.03), and Heidelberg (6% to 30%, p<0.01). Significant increases were identified for resistance to ciprofloxacin (p<0.05), nalidixic acid (p<0.01), sulfamethoxazole (p<0.01), tetracycline (p<0.01), and trimethoprim/sulfamethoxazole (p<0.01). Cephalosporin resistance increased, although not significantly (p = 0.06).

We suspected that these findings were confounded by serotype and therefore used logistic regression to model the odds of acquiring a resistant infection for each of the clinically important antimicrobial drugs (ampicillin, ceftriaxone, ciprofloxacin, gentamicin, or trimethoprim/sulfamethoxazole) as well as CIR. Serotype-adjusted log odds ratios were generated with year of infection entered as a discrete continuous variable. After adjusting for serotype, we found that with each subsequent year, patients were 30% more likely to acquire an infection that was resistant to quinolones (nalidixic acid or ciprofloxacin) and trimethoprim/sulfamethoxazole (Table 2). Resistance to ampicillin and cephalosporin also increased, although not significantly. We also found that with each year, odds of acquiring an infection with CIR increased by 13%.

**Table 2:** Serotype-adjusted odds of salmonellosis with resistance to specific antimicrobial drugs per year, Oregon, USA, 2004–2009*

Variable	Odds ratio (95% CI)†
Ampicillin	1.1 (1.0–1.1)
Cephalosporins	1.2 (1.0–1.6)
Gentamicin	1.0 (0.9–1.2)
Quinolones‡	**1.3 (1.1–1.5)**
Trimethoprim/sulfamethoxazole	**1.3 (1.1–1.5)**
CIR	**1.1 (1.0–1.2)§**

*Multiple logistic regression analysis of 2,127 isolates. Boldface indicates statistical significance at p<0.05. †Serotype adjusted; increased odds of resistance per year, 2004–2009. ‡Resistant to naladixic acid or ciprofloxacin. §p<0.01.

CIR was associated with hospitalization (odds ratio [OR] 1.5, 95% CI 1.1–2.0). This association was preserved after adjustment for serotype, patient age, patient race, and year (aOR 1.7, 95% CI 1.2–2.1). For patients with CIR infections, odds of invasive infection were increased, although not significantly, according to unadjusted or adjusted analyses (OR 1.4, 95% CI 0.9–2.2 and aOR 1.5, 95% CI 0.9–2.5, respectively).

### Risk Factors

Of the 2,127 patients included in the previous analyses, 1,813 (84.2% of all Oregon patients with NTS) were interviewed. For 305 (16.8%) of these patients, isolates had CIR, and for the remaining 1,508 (83.2%), isolates were susceptible to all clinically important antimicrobial drugs (Figure). Of the 1,508 isolates susceptible to clinically important drugs, 1,002 (55.3%) were susceptible to all drugs screened and 506 (27.9%) were resistant to at least 1 non-CIR drug.

According to the unadjusted analysis, several serotypes were more likely than the referent serotype, Enteritidis, to be resistant to >1 clinically important antimicrobial drug (Table 3). Patient sex, race, age, and ethnicity were not significantly associated with resistance.

**Table 3:** Associations of salmonellosis with CIR, Oregon, 2004–2009*

Variable	No. patients	% CIR isolates	Odds ratio (95%CI)		Adjusted odds ratio(95% CI)
Patient travel history					
No international travel	1,571	16.2	Referent		
Travel to Asia	**46**	**39.1**	**3.3 (1.8–6.1)**		**5.2 (2.6–10.4)**
Case type					
Sporadic	1407	18.6	Referent		
Outbreak	**406**	**10.8**	**0.5 (0.4–0.8)**		**0.5 (0.4–0.7)**
Year (odds of CIR cases/y)	**1,813**	**16.8**	**1.0 (0.9–1.1)**		**1.1 (1.0–1.2)**
*Salmonella* serotype					
Enteritidis	334	6.0	Referent		
Typhimurium	**260**	**25.0**	**5.2 (3.1–8.9)**		**6.2 (3.6–10.7)**
Heidelberg	**149**	**27.5**	**6.0 (3.3–10.6)**		**7.4 (4.1–13.5)**
Typhimurium var. Copenhagen	**92**	**53.3**	**17.9 (9.7–32.9)**		**20.2 (10.7–38.0)**
Newport	**82**	**41.5**	**11.1 (5.9–20.9)**		**10.8 (5.6–20.5)**
I 4, 5, 12:i:-	**79**	**19.0**	**3.7 (1.8–7.6)**		**4.1 (2.0–8.7)**
Montevideo	72	4.2	0.7 (0.2–2.4)		0.8 (0.2–2.7)
Saintpaul	**52**	**15.4**	**2.9 (1.2–6.9)**		**3.4 (1.4–8.3)**
Paratyphi B var. L+ Tartrate+	**50**	**24.0**	**5.0 (2.2–10.9**		**5.8 (2.6–13.1)**
All other	643	9.0	1.6 (0.9–2.6)		1.5 (0.9–2.5)
Patient age, y					
18–64	999	16.0	Referent		
<1	122	18.9	1.2 (0.8–2.0)		1.4 (0.9–2.4)
1–4	220	16.4	1.0 (0.7–1.5)		0.8 (0.5–1.2)
5–17	279	20.1	1.3 (0.9–1.8)		1.0 (0.7–1.5)
>65	193	15.5	1.0 (0.6–1.5)		0.9 (0.6–1.4)
Patient race					
White	1,662	16.7	Referent		
Not white	151	18.5	1.1 (0.7–1.8)		1.0 (0.6–1.6)

*Multiple logistic regression analysis of 1,813 patients; CIR, clinically important resistance to >1 of the following: ampicillin, ceftriaxone, ciprofloxacin, gentamicin, or trimethoprim/sulfamethoxazole. Boldface indicates statistical significance at p<0.05.

CIR was not associated with any other demographic risk factors or high-risk food or animal exposures. However when international travel was examined by individual countries or applicable United Nations region, CIR was significantly associated with travel to Southeast Asia (*24*). Associations of resistance with travel to Mexico and eastern Asia also approached significance (Table 4). The most common travel destinations in Asia where resistant infections were acquired were Thailand, China, and Malaysia/Indonesia. On the basis of these findings, we analyzed international travel by 3 destinations: Central America, including Mexico (135 patients), eastern and Southeast Asia (46 patients), and all other international travel destinations (77 patients).

**Table 4:** Unadjusted associations of CIR of *Salmonella* isolates among 1,813 patients, by travel destination, Oregon, 2004–2009*

Destination	No. patients	% CIR isolates	Odds ratio (95% CI)
None	1,571	16.8	Referent
Mexico	119	9.2	0.5 (0.3–1.0)
**Southeast Asia**	**29**	**41.4**	**3.5 (1.7–7.4)**
Europe	25	16.0	0.9 (0.3–2.8)
East Asia	17	35.3	2.7 (1.0–7.4)
Caribbean	16	12.5	0.7 (0.2–3.1)
Central America†	16	6.3	0.3 (0.1–2.5)
Africa	10	20.0	1.2 (0.3–5.9)
Oceania	5	20.0	1.2 (0.1–11.1)
Canada	5	40.0	3.3 (0.5–19.8)
Any travel	242	16.9	1.0 (0.7–1.4)

*CIR, clinically important resistance to >1 of the following: ampicillin, ceftriaxone, ciprofloxacin, gentamicin, or trimethoprim/sulfamethoxazole. Boldface indicates statistical significance at p<0.05. †Excludes Mexico.

Patients who were part of identified outbreak clusters were significantly less likely than patients with sporadic infections to have a resistant infection (OR 0.5, 95% CI 0.4–0.8). During our study period, 131 outbreaks (406 cases) occurred, and for 25 of these outbreaks a causative vehicle was successfully identified. To assess whether oversampling of cases from outbreak clusters could explain this association, we first restricted cases to 1 isolate per outbreak where a causative vehicle was implicated while retaining all cases from outbreaks for which a vehicle was not implicated (302 cases). Second, we further restricted cases to 1 isolate per outbreak, regardless whether a vehicle was identified (131 cases). In each of these analyses the magnitude, direction, and significance of the association was preserved (OR 0.6, 95% CI 0.4–0.8 and OR 0.5, 95% CI 0.3–0.9, respectively), suggesting that oversampling could not have explained this association. Furthermore, 53.6% of outbreaks had intra-outbreak cases for which the antimicrobial drug susceptibility profiles of the isolates differed.

The resultant main-effects model included the fixed variables of serotype, patient age, year of onset, and patient race, along with travel to eastern or Southeast Asia, and outbreak association (Table 3). We hypothesized that effect modification occurred between the variables of outbreak cases and travel to eastern or Southeast Asia, as well as between travel to eastern or Southeast Asia, serotypes, and outbreaks. However, no significant interactions at the p<0.25 level were identified. No other variables or exposures were significantly associated with CIR. This model had excellent goodness-of-fit (p = 0.87). The association between resistance and travel to eastern or Southeast Asia was preserved after exclusion of all outbreak-associated cases (aOR 4.6, 95% CI 2.3–9.4; Table 5). Similarly, the association between resistance and outbreak-associated cases was preserved after exclusion of patients with a history of international travel (aOR 0.5, 95% CI 0.4–0.8; Table 6). Therefore, these results suggest that inclusion of patients with a history of travel to Asia, as well as patients with outbreak-associated infections for the main analysis, was appropriate.

**Table 5:** Associations of salmonellosis with CIR among 1,407 sporadic cases only, Oregon, 2004–2009*

Variable	No. patients	% CIR isolates	Odds ratio (95% CI)	Adjusted odds ratio (95% CI)
Patient travel to Asia				
No	1,363	17.9	Referent	
Yes	**44**	**38.6**	**2.9 (1.6–5.4)**	**4.6 (2.3–9.4)**
Year ( odds of CIR cases/y)	**1,407**	**18.6**	**1.0 (0.9–1.1)**	**1.1 (1.0–1.2)**
*Salmonella s*erotype				
Enteritidis	254	7.5	Referent	
Typhimurium	**179**	**31.8**	**5.8 (3.3–10.2)**	**6.4 (3.6–11.5)**
Heidelberg	**94**	**33.0**	**6.1 (3.2–11.5)**	**6.9 (3.6–13.2)**
Typhimurium var. Copenhagen	**81**	**54.3**	**14.7 (7.8–27.9)**	**17.3 (8.9–33.4)**
Newport	**78**	**42.3**	**9.1 (4.7–17.3)**	**9.6 (4.9–18.6)**
I 4, 5, 12:i:-	**60**	**23.3**	**3.8 (1.8–8.0)**	**4.2 (1.9–9.0)**
Montevideo	48	2.1	0.3 (0.0–2.0)	0.3 (0.0–2.2)
Saintpaul	35	14.3	2.1 (0.7–5.9)	2.3 (0.8–6.7)
Paratyphi B var. L+ Tartrate+	**36**	**19.4**	**3.0 (1.2–7.7)**	**3.2 (1.2–8.5)**
All other	542	9.2	1.3 (0.7–2.2)	1.2 (0.7–2.1)
Patient age, y				
18–64	785	17.7	Referent	
<1	94	21.3	1.3 (0.7–2.1)	1.6 (0.9–2.8)
1–4	156	18.0	1.0 (0.6–1.6)	0.7 (0.4–1.2)
5–17	209	21.1	1.2 (0.8–1.8)	0.9 (0.6–1.4)
>65	163	18.4	1.0 (0.7–1.6)	1.1 (0.7–1.7)
Patient race				
White	1,295	18.1	Referent	
Not white	112	24.1	1.4 (0.9–2.3)	1.3 (0.8–2.2)

*Multiple logistic regression analysis. CIR, clinically important resistance to >1 of the following: ampicillin, ceftriaxone, ciprofloxacin, gentamicin, or trimethoprim/sulfamethoxazole. Boldface indicates statistical significance at p<0.05.

**Table 6:** Associations of salmonellosis with CIR for 1,571 patients, excluding patients with history of international travel, Oregon, 2004–2009*

Variable	No. patients	% CIR isolates	Odds ratio (95% CI)	Adjusted odds ratio (95% CI)
*Salmonella s*erotype				
Enteriditis	232	4.3	Referent	
Typhimurium	**245**	**22.5**	**5.6 (2.8–11.5)**	**6.9 (3.4–14.0)**
Heidleberg	**147**	**27.2**	**6.9 (3.3–14.6)**	**9.1 (4.3–19.0)**
Typhimurium var. Copenhagen	**85**	**52.9**	**19.7 (8.9–43.4)**	**26.0 (12.0–56.4)**
Newport	**66**	**48.5**	**16.1 (7.0–36.8)**	**19.3 (8.7–43.1)**
I 4, 5, 12:i:-	**77**	**18.2**	**3.3 (1.4–8.0)**	**5.1 (2.2–12.1)**
Montevideo	70	4.3	0.7 (0.2–2.8)	1.0 (0.3–3.8)
Saintpaul	**43**	**18.6**	**5.8 (2.0–16.7)**	**5.5 (2.0–15.1)**
Paratyphi B var. L+ Tartrate+	**45**	**26.7**	**6.3 (2.5–16.1)**	**9.0 (3.5–22.6)**
All other	561	8.0	1.6 (0.8–3.3)	1.8 (0.9–3.6)
Case type				
Sporadic	1190	18.7	Referent	
Outbreak	**381**	**11.0**	**0.6 (0.4–0.8)**	**0.5 (0.4–0.8)**
Year ( odds of CIR cases/y)	**1571**	**16.8**	**1.1 (1.0–1.2)**	**1.1 (1.0–1.2)**
Age, y				
18–64	817	16.5	Referent	
<1	119	18.5	1.1 (0.7–1.9)	1.3 (0.8–2.3)
1–4	204	16.2	0.9 (0.6–1.4)	0.8 (0.5–1.2)
5–17	245	18.8	1.1 (0.8–1.6)	0.9 (0.6–1.4)
>65	186	15.1	0.8 (0.5–1.3)	0.8 (0.5–1.4)
Race				
White	1662	16.7	Referent	
Not white	151	18.5	1.2 (0.7–1.9)	1.1 (0.7–1.8)

*Multiple logistic regression analysis. CIR, clinically important resistance to >1 of the following: ampicillin, ceftriaxone, ciprofloxacin, gentamicin, or trimethoprim/sulfamethoxazole.clinically important resistance. Boldface indicates statistical significance at p<0.05.

Patients with a history of recent travel to eastern or Southeast Asia were >5 times more likely to acquire a CIR infection than were patients with no history of recent international travel. The most common serotypes acquired among persons with a history of travel to Asia were Enteriditis (n = 13, 54% CIR), Typhimurium (n = 5, 60% CIR), Newport (n = 4, 25% CIR), I 4, 5, 12:i:- (n = 4, 50% CIR), Stanley (n = 3, 33% CIR), and Typhimurium var. Copenhagen (n = 2, 100% CIR). Patients with outbreak-associated infections were half as likely as those with sporadic infections to have CIR (Table 3).

To identify risk factors for resistance to individual antimicrobial drugs, we constructed models with each of the clinically important antimicrobial drugs. Travel to eastern or Southeast Asia was significantly associated with resistance to ampicillin, quinolones (nalidixic acid or ciprofloxacin), and trimethoprim/sulfamethoxazole (Table 7). Only individual serotypes were associated with resistance to cephalosporins or gentamicin, and no other risk factors were significantly associated with resistance to ampicillin, quinolones, or trimethoprim/sulfamethoxazole.

**Table 7:** Associations of salmonellosis with resistance to specific antimicrobial drugs and travel to Asia, Oregon, 2004–2009*

Drug	Adjusted odds ratio (95% CI)†
Ampicillin	**5.9 (2.9–11.8)**
Cephalosporins	1.0 (0.2–5.4)
Gentamicin	0.7 (0.1–5.3)
Quinolones‡	**22.0 (10.1–47.9)**
Trimethoprim/sulfamethoxazole	**4.5 (1.4–14.5)**

*Multiple logistic regression analysis for 1,813 patients, comparing odds of resistance for those with a history of travel to Asia with those with no history of international travel. Boldface indicates statistical significance at p<0.05. †Adjusted by serotype, year, patient age, patient race, and outbreak status. ‡Resistance to nalidixic acid or ciprofloxacin.

## Discussion

We found that NTS infections were more likely to have CIR with each subsequent year of our study. In Oregon during 2004–2009, the proportion of isolates susceptible to all antimicrobial drugs significantly decreased. Travel to eastern and Southeast Asia was associated with acquisition of *Salmonella* with CIR. Such travel was specifically associated with resistance to ampicillin, quinolones, and trimethoprim/sulfamethoxazole. Isolates from patients who were part of identified outbreak clusters were significantly less likely to be resistant, suggesting that resistance estimates based on outbreak cases alone may underestimate the true level of resistance. We also report that resistance is associated with increased hospitalization (*13*,*25*).

Our analysis was performed by using *Salmonella* susceptibility data from a surveillance system that captures ≈100% of confirmed infections, has antimicrobial drug susceptibility information for >95% of confirmed cases, and includes exposure histories for >84% of patients. This study is strengthened by having collected data on several known and potential confounders before the drug-susceptibility profiles were known. Our study design complements a previous National Antimicrobial Resistance Monitoring System/FoodNet study that analyzed antimicrobial drug resistance and increased disease severity (*13*). However, we determined where resistant infections were acquired (exposures) and patient outcomes associated with resistant infections by using an entire population. The ability to integrate resistance and serotype data with case-specific demographic and risk-factor data improves the generalizability and plausibility of our study and provides population-level risk estimates (*14*–*20*).

Widespread quinolone resistance in Southeast Asia has been reported (*26*); a better understanding of global use of antimicrobial drugs might suggest where resistant salmonellae are prevalent. Examination of serotype profiles among patients who had traveled to eastern or Southeast Asia and multivariate analyses adjusted for serotype provided strong evidence that the increased resistance in this region is widespread and not specifically attributable to a single serotype or regional serotype differences.

Increasing antimicrobial drug resistance has widespread implications for human health. We confirm the results of Varma et al. and Lee et al., who found antimicrobial drug resistance to be associated with increased likelihood of hospitalization (*13*,*25*). More severe infections can lead to treatment failure, sepsis, meningitis, and even death. If resistance to clinically important antimicrobial drugs continues to increase by 13% per year, as our data suggest, we can expect more severe illnesses, hospitalizations, and deaths, along with the accompanying higher economic costs.

The association between resistance and outbreak cases persisted after restricting the data in unadjusted and adjusted analyses. The lack of effect modification between outbreak cases and a history of travel to Asia in the multiple logistic regression modeling suggests that this finding is independent of travel. Resistant isolates might be less infectious and therefore less likely to cause recognizable outbreaks. Alternatively, common sources of resistant isolates might be less likely to cause widespread contamination.

Our study had limitations. We did not have information about previous antimicrobial drug use (*11*,*27*,*28*). However, this exposure would be expected to confound the observed associations nondifferentially, thereby resulting in lower point estimates. Reporting lags could have delayed risk-factor interviews, resulting in nondifferential recall bias. This bias would not be expected to explain the association between resistance and international travel and would ultimately lead to underestimation of the true effect size. Case ascertainment among persons with a history of travel to eastern or Southeast Asia could have been biased. This bias could have affected our analyses if more severe illness developed in travelers with resistant infections, who were more likely to seek health care or be reported than were travelers without resistant infections. However, according to a subanalysis, not presented here, we found that patients who traveled to eastern or Southeast Asia were less likely to be hospitalized than were those who had not recently traveled internationally (OR 0.4, 95% CI 0.2–1.2). This finding might be explained by a healthy traveler effect; thus, the association between resistance and travel to eastern and Southeast Asia cannot be explained by biased case ascertainment (*29*). The use of 2 laboratories for susceptibility testing could have resulted in systematic bias. Both laboratories were licensed and were using Clinical and Laboratory Standards Institute standardized methods, suggesting that this bias, if present, would be minimal and could not explain the observed associations.

This study demonstrates that antimicrobial drug resistance among NTS is increasing and has clinical and public health implications. Our analyses elucidated that travel to Asia is strongly associated with antimicrobial drug resistance. When considering antimicrobial drug therapy, providers should evaluate patient travel history and *Salmonella* serotype. Our results highlight the need for enhanced domestic surveillance for antimicrobial drug resistance and suggest a need for increased prudence regarding the use of antimicrobial drugs.
